# Cognitive processing speed improvement after cochlear implantation

**DOI:** 10.3389/fnagi.2024.1444330

**Published:** 2024-09-17

**Authors:** Isabelle Mosnier, Joël Belmin, Domenico Cuda, Raquel Manrique Huarte, Mathieu Marx, Angel Ramos Macias, Riad Khnifes, Ohad Hilly, Roberto Bovo, Chris J. James, Petra L. Graham, Paula Greenham

**Affiliations:** ^1^Unité Fonctionnelle Implants Auditifs, ORL, GH Pitié-Salpêtrière, AP-HP Sorbonne Université and Université Paris Cité, Institut Pasteur, AP-HP, Inserm, Fondation Pour l’Audition, Institut de l’Audition, Paris, France; ^2^Sorbonne Université and Hôpital Charles Foix, Paris, France; ^3^Ospedale Guglielmo da Saliceto, University of Parma, Piacenza, Italy; ^4^Clinica Universitaria de Navarra, Pamplona, Spain; ^5^Hôpital Purpan, CHU, Toulouse, France; ^6^Complejo Hospitalario Universitario Insular Materno Infantil, Las Palmas de Gran Canaria, Spain; ^7^Bnai Zion Medical Center, Haifa, Israel; ^8^Rabin Medical Center, Petah Tikva, Sackler Faculty of Medicine, Tel Aviv University, Israel; ^9^Azienda Ospedaliera di Padova, Padova, Italy; ^10^Cochlear France SAS, Toulouse, France; ^11^School of Mathematical and Physical Sciences, Macquarie University, Sydney, NSW, Australia; ^12^Greenham Research Consulting Ltd., Ashbury, United Kingdom

**Keywords:** cognition, elderly, cochlear implant, healthy aging, decline

## Abstract

**Background:**

Untreated hearing loss has an effect on cognition. It is hypothesized that the additional processing required to compensate for the sensory loss affects the cognitive resources available for other tasks and that this could be mitigated by a hearing device.

**Methods:**

The impact on cognition of cochlear implants (CIs) was tested in 100 subjects, ≥60 years old, with bilateral moderately-severe to profound post linguistic deafness using hearing aids. Data was compared pre and 12 and 18 months after cochlear implantation for the speech spatial qualities questionnaire, Mini Mental State Examination (MMSE), Trail making test B (TMTB) and digit symbol coding (DSC) from the Wechsler Adult Intelligence Scale version IV and finally the timed up and go test (TUG). Subjects were divided into young old (60–64), middle old (65–75) and old old (75+) groups. Cognitive test scores and times were standardized according to available normative data.

**Results:**

Hearing significantly improved pre- to post-operatively across all age groups. There was no change post-implant in outcomes for TMTB, TUG or MMSE tests. Age-corrected values were within normal expectations for all age groups for the TUG and MMSE. However, DSC scores and TMTB times were worse than normal. There was a significant increase in DSC scores between baseline and 12-months for 60- to 64-year-olds (*t*[153] = 2.608, *p* = 0.027), which remained at 18 months (*t*[153] = 2.663, *p* = 0.023).

**Discussion:**

The improved attention and processing speed in the youngest age group may be a consequence of reallocation of cognitive resources away from auditory processing due to greatly improved hearing. The oldest age group of participants had cognition scores closest to normal values, suggesting that only the most able older seniors tend to come forward for a CI. Severe to profoundly deaf individuals with hearing aids or cochlear implants were still poorer than age-equivalent normally hearing individuals with respect to cognitive flexibility, attention, working memory, processing speed and visuoperceptual functions. Due to a lack of data for the TUG, TMTB and DSC in the literature for hearing impaired individuals, the results reported here provide an important set of reference data for use in future research.

## Introduction

Europe and Asia have the highest proportion of individuals over the age of 65 and the lowest birth rates. The Unites States is also approaching a similar demographic (Population Reference Bureau, 2019). Hearing loss and cognitive decline are two issues which face an aging population. Disabling hearing loss as defined by the World Health Organization (WHO), affects 6% of the world’s population, with the majority of this group being adults over the age of 65 (World Health Organization, 2021). Dementia prevalence is also thought to be around 6% for men and 8% for women over the age of 70 years (Nichols et al., 2022). Consequentially, age related hearing loss and Alzheimer’s are both top 10 contributors to the burden of disease for those aged 75 and over (Vos et al., 2020). Better understanding of the prevention and effective treatment of these two diseases is therefore of the utmost importance for global public health policy.

Furthermore, recent evidence has shown that hearing loss and cognitive impairment are related (Fuller-Thomson et al., 2022). Those with untreated hearing loss have significantly poorer cognition, with the magnitude of the deficit associated with the degree of hearing loss (Lin et al., 2011; Amieva et al., 2015; Taljaard et al., 2016). Thus, hearing loss is listed as the largest possible modifiable risk factor for dementia (Mukadam et al., 2019; Livingston et al., 2020). Although a link between cognitive decline and hearing loss seems to be clear, the mechanisms by which this occurs are not. There are a few hypotheses postulated for this, and multiple factors may be combined a detailed discussion can be found in Tarawneh et al. (2022). The first theory postulates that both diseases share a common third-party cause. If this was solely the case, both diseases would be expected to progress regardless of any hearing loss treatment. The second theory is that sensory deprivation resulting in loss of input to the cortex causes the restructuring of auditory and cognitive systems, affecting both hearing and cognition. This theory would certainly lend itself to the restoration of hearing input being effective in halting progression, although effects are unlikely to be reversable. As an adjunct to this, a third proposed mechanism includes an interaction between the altered auditory cognition due to hearing loss and Alzheimer’s disease (Griffiths et al., 2020). The third theory is that cognitive load is increased by the additional processing required to compensate for the sensory loss resulting in less available capacity for other cognitive processes. It is hypothesized that if this cognitive load could be reduced with corrective amplification such as a hearing aid or cochlear implant, functions such as cognitive processing speed and ability may be restored. However, current evidence to support this hypothesis is mixed. Finally, the loss of social interaction and increased loneliness associated with hearing loss may increase or accelerate cognitive decline (Cuda et al., 2024).

A meta-analysis on the use of hearing restorative devices showed they were associated with a 19% decrease in the hazard of long-term cognitive decline over a duration ranging from 2 to 25 years, with a 3% improvement in cognitive test scores in the short term (Yeo et al., 2022). Although evidence in a review by Carasek et al. (2022) supports the conclusions of Yeo et al. (2022) and Yang et al. (2022) found no significant effects of hearing devices on cognitive decline (Carasek et al., 2022; Yang et al., 2022). A recent controlled randomized study showed that fitting hearing aids reduced cognitive impairment in a subgroup of older adults at increased risk for cognitive decline and with lower baseline cognitive function (Lin et al., 2023). However, the hearing aid did not reduce 3-year cognitive decline for the total cohort. Additionally, a large prospective longitudinal cohort study showed that hearing aid users had significantly better cognitive performance at 3 years post-fitting than a control group of older adults with untreated hearing loss or normal hearing (Sarant et al., 2024).

Cochlear implants (CI) can restore hearing input for those with bilateral severe to profound hearing impairment when hearing aids are no longer sufficient. As well as enhanced speech recognition and ability to communicate verbally, they improve quality of life and reduce loneliness and hearing handicap (Cuda et al., 2024). What is currently not clear is if CIs can bring additional gains in cognition to those who are already wearing hearing aids. Research specifically focused on CIs has provided mixed evidence of their cognitive benefits (Claes et al., 2018). Huge variation exists in the tests used in different studies and small groups or sub-groups of subjects were used in many analyses, which were not powered to look at specific cognitive outcomes. Individual studies have shown some cognitive benefits of CIs in a range of areas and tests such as spatial working memory, attention and cognitive flexibility, word list tasks, clock drawing, inhibition and recall and verbal fluency (Mosnier et al., 2015; Jayakody et al., 2017; Sarant et al., 2019; Issing et al., 2021; Mertens et al., 2021). However, no consensus exists and any evidence that improving hearing with a CI has additional benefits for cognition are limited (Claes et al., 2018; Hamerschmidt et al., 2023).

Here we analyze data from the study described by Marx et al. (2020) which monitored a variety of healthy-aging domains including hearing ability, physical and mental health and cognition in a large prospectively recruited cross-cultural sample of adults aged ≥60 years old pre- and post-cochlear implantation. Results on functional outcomes were reported in Cuda et al. (2024). The primary hypothesis explored in the current paper was that providing CI treatment in the elderly would improve cognition, compared to the preimplant condition. The effect of CIs was studied in the context of normal cognition scores and those of hearing-impaired individuals with hearing aids.

## Materials and methods

This was an observational repeated-measures, single-subject, study where each subject acts as his/her own control. Subjects ≥60 years old with bilateral post linguistic onset of moderately severe to profound deafness, who met all local criteria for unilateral cochlear implantation, were recruited and evaluated as part of their routine clinical visits. Recruitment was from November 2017 to March 2022. Implant clinics of multiple nationalities were chosen for their experience and existing capacity to recruit and treat elderly CI candidates within a reasonable time frame for the study. All subjects who had been assessed as suitable for a CI and had already decided to proceed with a CI manufactured by Cochlear Ltd. and met the study criteria were invited to participate. Full criteria for study participation are given in the broader study protocol (Marx et al., 2020). All enrolled subjects independently gave their written informed consent for participation in the study and ethics approval was given by the Comitato Etico (AVEN) Area Vasta Emilia Nord, Piacenza, Italy.

### Measures

Subjects were assessed in a variety of domains both before and at 12 and 18 months post-implantation. The protocol allowed for a variation of 1 month from the scheduled follow up session. Baseline values were collected less than 2 months before surgery. Due to the multi-lingual nature of the study, speech perception measures for the group could not be combined. Therefore, subjective hearing performance was assessed using the speech spatial qualities questionnaire (SSQ). This is a self-assessment scale comprised of 49 questions divided into three subcategories: speech (comprehension), spatial (hearing in space) and quality (speech and sounds) (Gatehouse and Noble, 2004). Each question is scored on a 10-point rating scale, with higher numeric values reflecting greater ability for the responder. A clinically significant difference on this measure is set at a rating change of 1.0 between test intervals for each overall subcategory score (Noble and Gatehouse, 2006).

Cognition was assessed using four cognitive tests:

(1) Mini Mental State Examination (MMSE) is a 30-point screening test used to estimate the severity and progression of cognitive impairment, and to follow the course of cognitive changes in an individual over time (Folstein et al., 1975). The MMSE examines functions including, attention, calculation, recall, language, orientation and ability to follow simple commands. A cut off score of 24 to indicate normal function was used with a sensitivity of 0.85 and a specificity of 0.9 (Creavin et al., 2016).
(2) Digit-Symbol-Coding (termed Coding here) from the Wechsler Adult Intelligence Scale version IV (WAIS- IV) (Wechsler, 2008) is a neuropsychological test sensitive to brain damage, dementia, age and depression, primarily assessing processing speed. It consists of nine digit-symbol pairs followed by a list of digits. Under each digit the subject should write down the corresponding symbol as fast as possible. The number of correct symbols within the allowed 120 s is measured. Coding shows a strong decline with age.
(3) Trail making test B (TMTB) is a neuropsychological test assessing executive function requiring skills of attention, concentration, processing speed and mental flexibility (Reitan and Wolfson, 1985). This test consists of 25 circles distributed over a sheet of paper. The circles include both numbers (1 – 13) and letters (A – L); the subject draws lines to connect the circles in an ascending pattern, with the task of alternating between the numbers and letters (i.e., 1-A-2-B-3-C, etc.). The time the patient takes to connect the “trail” is their score.
(4) Balance and cognition were assessed using the timed up and go test (TUG). This measures the time (in seconds) a person takes to stand up from a standard armchair, walk three meters (i.e., 10 feet), turn around, walk back to the chair, and then sit down again (Podsiadlo and Richardson, 1991). The TUG test is associated with global cognition and executive function (Donoghue et al., 2012). Poor TUG scores are associated with an increased risk of future dementia occurrence (Lee et al., 2018). The TUG also assesses balance. One of the complications associated with cochlear implantation is disruption of the vestibular system and temporarily impaired balance (Colin et al., 2018). This could have a greater impact on those who are elderly and at risk of falls.

Subjects were evaluated in their native languages (Italian, French, Spanish, Arabic or Hebrew). Certificated forwards/backwards translation was carried out by external professional translation providers. Written instructions as well as verbal instructions were provided to all participants for all the tests, to mitigate the effects of hearing loss on the understanding of the task.

Normative data for the cognitive measures was taken from the relevant manuals and publications. The Trail making test and symbol coding task are relatively new clinical measures for the hearing-impaired population. Therefore, a systematic review was conducted to identify data for these measures for hearing impaired individuals using a hearing aid or CI in order to provide comparative data for this study. A PubMed search was conducted on 18/09/23 using the search string: (Cognition[Title/Abstract] OR “Cognitive decline” OR “Cognitive impairment” AND (“Hearing treatment” OR “hearing aid” OR “hearing aids” OR “cochlear implant” OR “cochlear implants” OR “cochlear implantation”) AND ((english[Filter]) AND (2013:2023[pdat]))) NOT (children[Title/Abstract] OR pediatric[Title/Abstract] OR paediatric[Title/Abstract]) Filters: English, from 2013 - 2023 Sort by: Publication Date. 167 studies were identified, of which 112 were relevant and went forward to full text assessment. Forty studies reported digit symbol coding or TMTB scores and went forward for further review. Studies were included for comparison where data was reported for a sample where all the subjects in the cohort used a hearing aid or a CI and the raw values were specified for the symbol coding test from the WAIS- IV or TMT-B. Hearing loss was required to be measured using a pure tone audiogram and not to be self-reported. Studies with duplicate or overlapping samples were excluded.

### Statistical methods

Cognitive measures Digit-Symbol-Coding, TMTB and MMSE, and TUG times were standardized (converted to z-scores) according to available norms based on age group, education level and sex.

MMSE scores were reversed and then log transformed according to the method of Huppert et al. (2005). Transformed scores were standardized according to age and gender based on mean and standard-deviation interval data from Table 1 in Huppert et al. (2005).

**Table 1:** Characteristics of the 98 subjects included at baseline.

Characteristics		Count (%)
Sex	Female	43 (44.4)
	Male	55 (55.6)
Implant side	Left	32 (32.7)
	Right	66 (67.3)
Hearing loss type in implanted ear	Mixed	7 (7.1)
	Sensorineural	91 (92.9)
Hearing loss onset in implanted ear	Progressive	82 (83.7)
	Sudden	15 (15.3)
	Congenital (post-lingual)	1 (1)
Hearing loss severity in implanted ear (as per ASHA guidelines)	Moderate	2 (2.0)
	Severe	27 (27.6)
	Profound	69 (70.4)
Etiology	Unknown	60 (61.2)
	Otosclerosis	9 (9.2)
	Chronic Otitis Media	7 (7.1)
	Meniere’s	6 (6.1)
	Other	5 (5.1)
	Genetic	3 (3.1)
	Trauma	3 (3.1)
	Noise Exposure	2 (2.0)
	Ototoxic Drugs	2 (2.0)
	Meningitis	1 (1.0)
Pre-implant hearing aid (HA) use	Bilateral	70 (71.4)
	Left hand side	12 (12.2)
	Right hand side	10 (10.2)
	No HA	6 (6.2)
Highest level of education	Post secondary/tertiary	55 (56.1)
	Secondary education	23 (23.4)
	Primary education	17 (17.3)
	Pre-primary education	3 (3.1)
Current work status	Retired	76 (77.6)
	Working full time	10 (10.2)
	Working part time	6 (6.1)
	Voluntary not employed	3 (3.1)
	Other	3 (3.1)

ASHA, American Speech-Language-Hearing Association.

TUG times were transformed according to age-range using obtained data from Table 2 reported in the meta-analysis by Bohannon (2006). Standard deviations for the normative data by age-range were computed from 95% confidence limits and sample sizes. One data point with a TUG of 460 s was removed from the analysis as it exceeded the plausible time frame for the task.

**Table 2:** Main effects and interaction effects.

	Time point			Age group			Time-point × age-group		
	DF	*F*	*p*	DF	*F*	*p*	DF	*F*	*p*
SSQ	2/163.2	63.21	**<0.001**	2/92.7	0.2110	0.810	4/163.5	0.3996	0.809
MMSE	2/158.7	0.1886	0.828	2/87.1	2.3385	0.103	4/159.1	0.7809	0.539
Coding	2/156.4	1.6397	0.197	2/92.7	2.6295	0.078	4/156.6	2.6256	**0.037**
TMTB	2/146.4	1.9424	0.147	2/87.0	4.5277	**0.013**	4/146.6	0.4195	0.794
TUG	2/160.2	1.7035	0.185	2/90.2	0.7557	0.473	4/160.5	1.5350	0.195

Data taken from linear mixed model analyses of SSQ total scores and standardized cognitive outcome measures. *p*-values <0.05 are in bold.

TMTB times were log transformed (Gurgel et al., 2022). Age and education level means and standard deviations for log transformed times were obtained by fitting probability density functions to the log transformed decile data, provided in Tombaugh (2004).

Standardized WAIS-IV digit-symbol coding scores were obtained by looking up raw scores in tables by age-range provided in the WAIS-IV Technical and Interpretive Manual (Wechsler, 2008).

Transformed z-scores were analyzed using a linear mixed effects model. All available data was used for visits and age groups, together with their interaction, as fixed effects. Missing data is assumed to be missing at random, i.e., the reason for being missing does not relate to the outcome measure.

A visual inspection of normal quantile plots was included to assess the normality of the errors and random effects. Tukey pairwise comparisons were used to compare all pairs of time points. A 5% significance level was used throughout.

## Results

### Subjects

One hundred subjects were originally recruited. However, two subjects were identified post-hoc as not meeting inclusion/exclusion criteria and were excluded from the analysis. Characteristics of the 98 subjects are outlined in Table 1.

Seven subjects had no follow-up data. Reasons given for this included loss to follow-up (*n* = 3), protocol deviation (*n* = 2), consent withdrawn (*n* = 1), and investigator decision (*n* = 1).

Mean age (standard deviation, SD) was 71.7 (7.6) (range 60–91) years, mean age at onset of severe hearing loss was 65.2 (12.3) (range 9–88) years. Duration of severe to profound hearing loss was a mean of 7.2 (10.2) years with a median of 3 years. Three subjects reported having two native languages and 42 reported being fluent in at least one other language. Subjects reported typically healthy lifestyles with 86% non-smokers, 70% reporting drinking 4 times a month or less and 70% active daily or weekly with most taking up to 3 h of gentle exercise per week. Mean body mass index (SD) was 26 (4.3) (range 17–41), indicating an overweight cohort, on average. Scores on the Geriatric Depression Scale-15 indicated that 90% had either no (72%) or mild (18%) depression.

### Subjective hearing performance

SSQ total scores varied significantly with time point, but not by age group, with no significant interaction (Table 2). Mean SSQ total scores increased significantly at 12-months (*t*[164] = 9.27, *p* < 0.001), with no significant further increase to 18-months (*t*[163] = 0.757, *p* = 0.730). Hearing performance also improved in all three sub domains. Paired comparisons indicated that all scales improved statistically and clinically significantly from baseline to 12 months and 18 months (all *p* < 0.001 for Speech and Spatial, and *p* < 0.01 for Qualities), but not between 12 and 18 months (Table 3).

**Table 3:** Raw scores for the Speech Spatial Qualities questionnaire.

Scale	Baseline *n* = 97 Mean (SD)	12 months *n* = 85 Mean (SD)	18 months *n* = 82 Mean (SD)
Speech	1.96 (1.55)	3.87 (2.13)	4.19 (2.04)
Spatial	2.78 (2.04)	4.53 (2.2)	4.70 (2.17)
Qualities	3.57 (2.14)	5.25 (2.19)	5.41 (2)
Total	2.82 (1.75)	4.6 (2)	4.81 (1.9)

Each of the three sub scales are shown along with the total score. Maximum score is 10, representing subjectively perfect hearing. SD, standard deviation.

### Cognitive tests

Subjects were divided into the same three age groups used in the previous publication with young old defined as 60–64 years, based on the United Nations definition of old, middle old (65–75 years) and old old (75+ years) (Cuda et al., 2024). Age was not considered as a continuous variable due to the possibility of non-linear age effects and poor distribution of age across the sample.

Table 4 reports raw score summaries for all the outcome measures, overall and by age group to serve as a reference. Scores on both the TMTB and the WAIS IV coding test are affected by education level and/or age. Consequently, scaling individual scores by the mean value for age and education level allowed for accurate comparisons to be made across visits and within age groups. Transformed scores are also provided in Supplementary File 1 so that meaningful statistical comparisons can be made in the future.

**Table 4:** Cognition results reporting raw score summaries for each of the outcome measures overall and by age group at each time point.

Test	Baseline Mean (SD) [range]	12 months Mean (SD) (range)	18 months Mean (SD) range
Mini Mental State Examination	*N* = 96 26.8 (2.9) [17 – 30]	*N* = 84 26.9 (2.8) [20 – 30]	*N* = 83 27.1 (2.6) [20 – 30]
Age 60–64 (*n* = 20)	27.3 (2.9) [22 – 30]	26.8 (2.9) [22 – 30]	27.4 (2.5) [22 – 30]
Age 65–75 (*n* = 45)	27.0 (2.7) [21 – 30]	26.9 (2.8) [20 – 30]	27.3 (2.8) [20 – 30]
Age 75–93 (*n* = 31)	26.1 (3.1) [17 – 30]	27.1 (2.8) [21 – 30]	26.8 (2.4) [20 – 30]
WAIS-IV Symbol Coding Test	*N* = 91 39.8 (22.7) [6 – 120]	*N* = 81 40.2 (23.7) [4 – 101]	*N* = 82 42.6 (25.8) [0 – 104]
Age 60–64 (*n* = 19)	35.1 (15.4) [10 – 62]	44.1 (23.6) [11 – 98]	46.8 (24.6) [12 – 104]
Age 65–75 (*n* = 43)	40.3 (22.1) [6 – 107]	41.9 (24.2) [8 – 101]	43.2 (25.5) [8 – 97]
Age 75–93 (*n* = 29)	42.1 (27.3) [9 – 120]	35.1 (23.1) [4 – 86]	39.1 (27.3) [0 – 102]
Trail Making Test (TMTB)	*N* = 90 156 (86) [29 – 372]	*N* = 76 128 (69) [22 – 367]	*N* = 74 138 (83) [32 – 464]
Age 60–64 (*n* = 16)	144 (110) [29 – 372]	106 (46) [33 – 209]	95 (35) [32 – 151]
Age 65–75 (*n* = 44)	143 (74) [60 – 368]	121 (54) [38 – 274]	131 (73) [35 – 367]
Age 75–93 (*n* = 30)	182 (85) [48 – 327]	155 (92) [22 – 367]	174 (102) [53 – 464]
Timed Up and Go	*N* = 97 11.7 (7.1) [3 – 65]	*N* = 85 10.9 (4.5) [5 – 31]	*N* = 82 10.7 (4.4) [4 – 33]
Age 60–64 (*n* = 20)	9.6 (3.3) [6 – 17]	9.8 (3.8) [5 – 20]	9.3 (2.8) [5 – 16]
Age 65–75 (*n* = 45)	12.0 (8.7) [3 – 65]	10.0 (3.0) [5 – 19]	10.1 (2.8) [5 – 17]
Age 75–93 (*n* = 32)	12.5 (6.0) [4 – 35]	12.8 (6.0) [7 – 31]	12.4 (6.2) [4 – 33]

Commonly transformed scores are available in Supplementary File 1.

### MMSE

**Figure 1:**
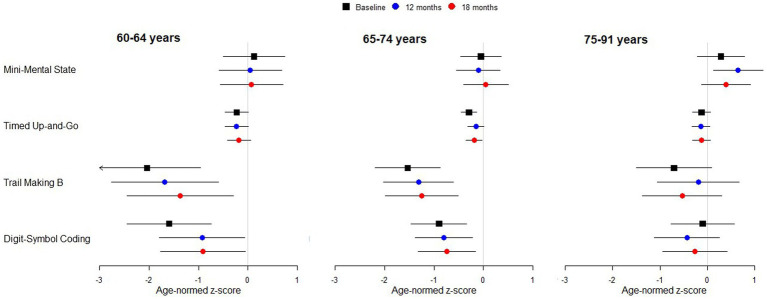
Standardized scores for cognitive test battery by age group. Points indicate estimated marginal means with 95% simultaneous confidence intervals. Arrowhead indicates the interval extends below −3. A confidence interval that includes zero indicates performance in line with expectation for normally hearing individuals of the same age. Negative scores indicate performance is worse than normal.

There was no statistically significant or clinically meaningful change in MMSE scores following implantation (Table 2). MMSE scores for the sample were comparable to normative values by age range (Figure 1). Baseline scores indicated that 75% of subjects recruited had no cognitive impairment at baseline with mean scores of 26.77 (SD 2.89) range 17–30 (Table 4).

### Digit symbol coding task

WAIS-IV coding scores standardized for age did not vary significantly across time points or by age group, however, there was a significant interaction between time-point and age-group (Table 2). Post-hoc comparisons revealed a significant increase in coding score between baseline and 12-months for 60- to 64-year-olds (*t*[153] = 2.608, *p* = 0.027, Figure 1). This increase remained stable to 18-months (*t*[153] = 2.663, *p* = 0.023). There was no increase in the other age groups.

Comparison to values for age equivalent normally hearing subjects showed that coding scores for the subjects in the 60–64 and 65–74 year age groups were statistically significantly below normal, both before and after implantation, with mean scores at least 0.5 standard deviations below normal (Figure 1). The 60–64 years group had the poorest coding score at baseline compared to the other age groups, with mean standardized scores nearly two standard deviations below the age equivalent normal values. After implantation, scores improved by almost one standard deviation.

**Figure 2:**
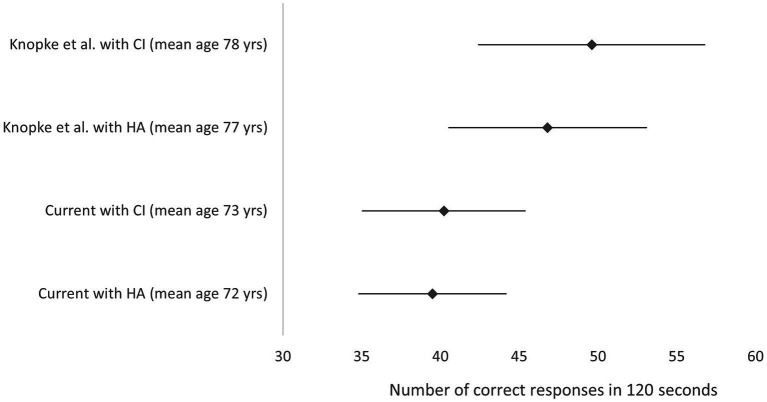
Mean symbol coding score and 95% confidence interval from the WAIS-IV. Data is represented for Knopke et al. (2021) study with HA before implantation and with the CI 12 months post- surgery and for the current study data. Higher scores indicate better performance. *Y* axis labels indicate study, device (mean age). HA, hearing aid; CI, cochlear implant; yrs., years.

The literature review conducted to identify other scores for the digit symbol coding data task for hearing impaired individuals identified only one paper where mean scores for the WAIS IV symbol coding task were reported in individuals using hearing aids or a CI (Figure 2; Knopke et al., 2021). Papers were excluded where the WAIS-R was used, where the time allocated for the coding test was 90 s. The WAIS III used 90 or 120 s for responses, however, the WAIS IV update made significant changes to the WAIS III digit symbol coding tasks, therefore comparisons to WAIS lll are also invalid (Ashendorf, 2012).

### Trail making test B

Standardized TMTB scores did not vary significantly across time points. However, they did differ by age group (Table 2). Standardized TMTB scores were better and closer to normal for the old-old 75–91 year group compared with the young, 60–64 years group (t-ratio [88.1] = 2.662, *p* = 0.027). Differences for other paired comparisons were not significant (oldest vs. middle, t-ratio [90.0] = 2.429, *p* = 0.051; youngest versus middle, t-ratio [88.7] = 0.789, *p* = 0.820). Furthermore, standardized TMTB times for the younger and middle groups were statistically significantly lower than the norm (z-score < 0, Figure 1).

**Figure 3:**
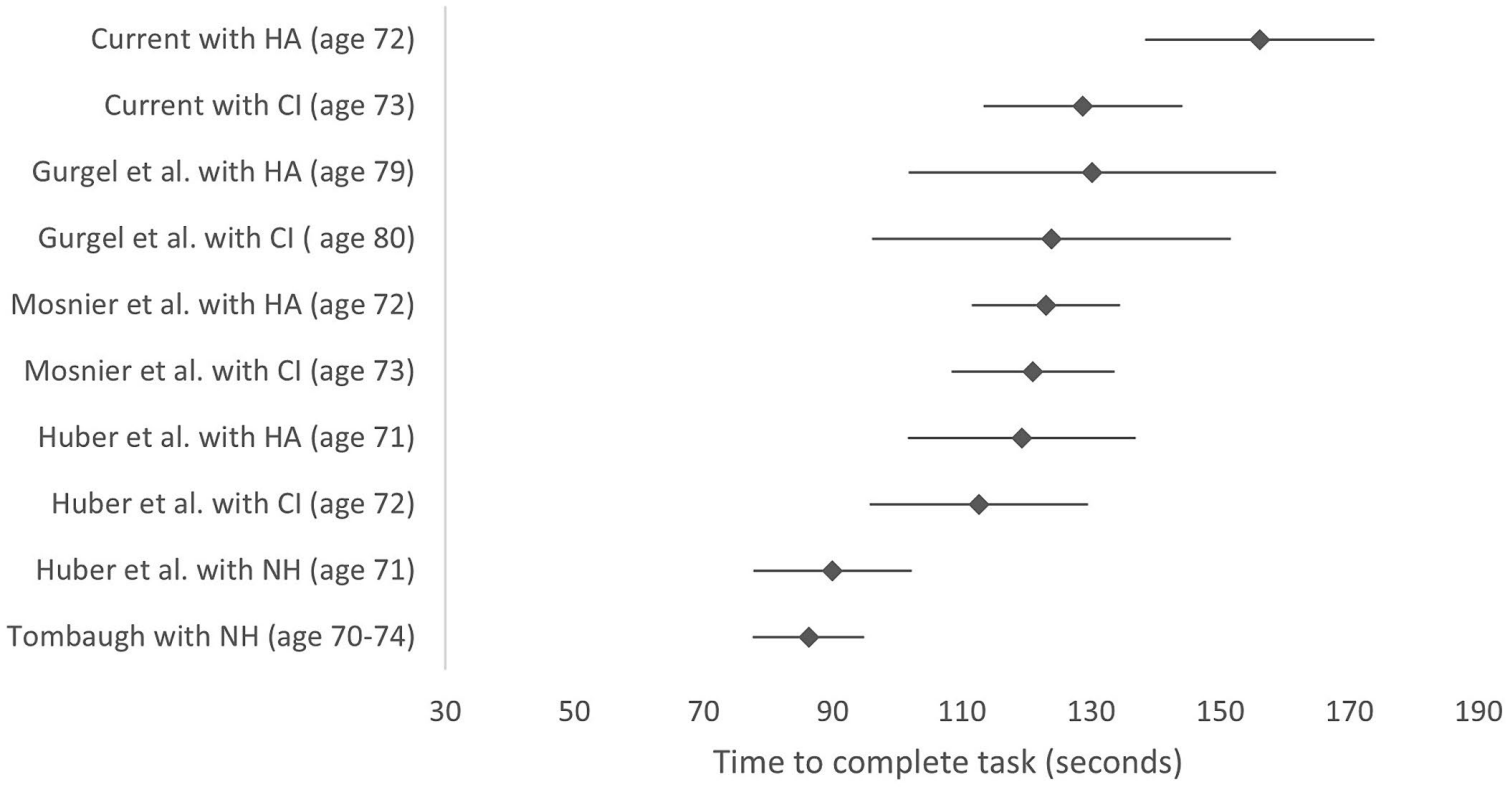
TMTB scores comparing current study with studies identified in the literature review. Lower scores indicate better performance. Scores are reported with hearing aid (HA) before implantation and with the cochlear implant (CI) at 12 months post-surgery. Mean values and 95% confidence intervals are shown. *Y* axis labels indicate study, device (mean group age in years). HA, hearing aid; CI, cochlear implant; NH, normally hearing.

The systematic literature review only identified four papers reporting TMTB scores where all the subjects in the cohort used a hearing aid or a CI, hearing loss had been measured using audiometry and the raw values were reported (Mosnier et al., 2018; Huber et al., 2021; Gurgel et al., 2022; Wu et al., 2022). The Huber et al. (2021) study also included a sample of normally hearing individuals as well as data on CI recipients before and after surgery. Wu et al. (2022) paper was later excluded because a computerized administration of the TMTB was used, making the test easier than the pen and paper version used here. A forest plot comparing these studies to the current data is shown in Figure 3.

Data for normally hearing subjects is also included from samples of normally hearing individuals from the Huber et al. (2021) study and the Tombaugh (2004) normative data for those aged 70–74 with at least 12+ years of education (Tombaugh, 2004; Huber et al., 2021).

The forest plot indicates that baseline TMTB scores with the hearing aid pre-CI surgery were higher (worse) for the current study than those reported for other cohorts in other studies, although postoperative CI scores were in line with previous reports.

### Timed up and go

Age-corrected standardized TUG times did not vary significantly with time point or age group, and there was no significant interaction between time point and age group (Table 2). TUG mean z-scores were consistently just below the expected normal value regardless of whether or not subjects were wearing hearing aids prior to implantation or a CI afterwards.

## Discussion

The subjective hearing performance reported via the SSQ showed a clinically and statistically significant improvement from baseline after CI implantation. Subjects’ subjective ability to hear speech, localize sounds and the overall quality of the sound increased from baseline to 12 months and 18 months, but did not improve further between 12 and 18 months. Improvements in hearing were independent of age group, with the old old reporting similar subjective improvements as the young old group.

At baseline, the study sample had high MMSE scores with 75% having no cognitive impairment. When compared to age-appropriate normal values, MMSE scores were in line with expectations from a random normal population sample (Huppert et al., 2005). This was not an unexpected result as CI criteria usually exclude those with chronic depression, dementia, and cognitive disorders and the number of subjects with any depression in this sample were low (Marx et al., 2020; Cuda et al., 2024). MMSE scores did not change significantly after implantation. This is comparable with other studies where no change on the MMSE was shown after 12 months of CI use (Mosnier et al., 2018; Huber et al., 2021; Gurgel et al., 2022). However, the MMSE was originally meant to be used as a quick screening tool for dementia in clinical settings and is known to be poor at detecting mild cognitive impairment (Baek et al., 2016).

The TUG test, assessing both balance and cognition, showed that the study population was approximately normal for their age and there was no change in scores between pre and post implant visits (Figure 1). This is in line with le Nobel et al. (2016) who did not find that balance, as measured with the TUG, was affected by the CI surgery (le Nobel et al., 2016). There is some evidence that those with hearing loss experience reduced balance function and scores were just below the expected norms for both hearing aids and the CI (Koh et al., 2015).

The literature review identified relatively few studies where TMTB or symbol coding tasks were reported for hearing impaired individuals. Thus, the results reported in Table 1 and the Supplementary File provide an important set of reference data for hearing aid and CI use. However, the sample only represent those severe to profoundly deaf hearing aid users who come forward for a CI and not the total population.

Analysis of the results of the TMTB and coding tests posed four main questions.

1. Why were coding scores worse than normal for the 60–64 and 65–75 age groups?

Coding scores in the young old and middle old age groups were poorer than for the normal hearing group, regardless of whether hearing aids or cochlear implants were used. The young old group had the poorest baseline coding scores of nearly two standard deviations below normal. The coding task is part of the WAIS-IV Processing Speed Subtest and consists of three measured abilities, visual-motor coordination, motor and mental speed, and visual working memory (Wechsler, 2008). Good performance on the coding test requires intact motor speed, good attention, and visuoperceptual functions, including scanning and the ability to write or draw (i.e., basic manual dexterity). Processing speed is also related to other measures of cognitive ability such as working memory, and performance may also be affected by other executive functions such as planning and strategizing (Finkel et al., 2007; Jaeger, 2018). One theory for the impact of hearing loss on cognition is that the excessive cognitive load dedicated to auditory perceptual processing may cause structural changes to the brain, diverting cognitive resources away from other cognitive processes (Tarawneh et al., 2022). In studies using the digit symbol substitution test, hearing loss was independently associated with poorer scores (Golub et al., 2020; Chern et al., 2022). The greater the hearing loss, the worse the score. Those with hearing loss also show a more rapid decline in digit symbol coding scores than those without (Uchida et al., 2012). Even mild-to-moderate acquired hearing loss may limit individuals’ ability to orient and divide attention and flexibly allocate attentional resources (Golub et al., 2020; Chern et al., 2022; Bonmassar et al., 2023).

2. Why might the implant improve coding scores for the young old group?

After implant, coding scores for the young old group improved statistically significantly at 12- and 18-months post-surgery by almost one standard deviation to values more in line with the 65–74-year-olds. There was no change in coding score pre and post implant for the middle old (65–74 years) and old old (75–91 years) age groups. Knopke et al. (2021) reported improved processing speed after implantation using the full test battery in the WAIS IV, but not for the symbol coding task in isolation (Knopke et al., 2021; Häußler et al., 2023). This could support the theory that the improved hearing provided by the CI released cognitive capacity, which could then be used to improve processing speed. It is possible that the young old group were still able to “bounce back” in a way that the middle and old old group no longer could. The younger cohort had the poorest baseline scores with their hearing aids and with the small sample sizes per group, it cannot be entirely discounted that the low scores pre-implantation for this group made it more likely that scores would increase (i.e., regression toward the mean).

3. Why is TMTB much worse than normal for 60–64 and 65–75 age groups?

TMTB scores were much worse in comparison to normal for both the young old and middle old groups both before and after implantation. Overall, TMTB scores for the whole group at baseline with the hearing aid were also worse than the scores reported in the other studies retrieved from the literature review. The impact of hearing loss on working memory is thought to be related to the allocation of cognitive resources, which are normally used for tasks such as storing auditory information into memory, and decoding the speech signal (Rönnberg et al., 2008). The ability to manipulate working memory rather than processing speed is thought to contribute most to TMTB performance and is particularly important when speech input is degraded, as is the case when using hearing aid or a CI (Sánchez-Cubillo et al., 2009; Rönnberg et al., 2013). Working memory is a buffer that holds memories accessible while a task is performed (Breedlove and Watson, 2019). It enables the listener to retain relevant information while listening to speech. However, the more ambiguous or degraded a stimulus is, the more working memory is needed to gather sufficient information for decision making (Rönnberg et al., 2013). Trail B performance in older individuals also measures the ability to shift attention to a new task (Sánchez-Cubillo et al., 2009). Thus, poor TMTB scores for older severely hearing-impaired individuals may be indictive of poorer cognitive flexibility (Huber et al., 2020). Scaled scores for the TMTB showed that there was no evidence of a change after implant. This is in line with the literature where most studies have reported no change in TMTB scores at 1 year post implant (Mosnier et al., 2018; Huber et al., 2021; Völter et al., 2022). Only Gurgel et al. (2022) showed a significant change in scaled mean TMTB scores (Gurgel et al., 2022). Improvements in overall working memory after implantation have been observed in other studies using direct measures such as the operation span task, spatial working memory and the working memory index from the WAIS-IV (Jayakody et al., 2017; Knopke et al., 2021; Völter et al., 2022; Häußler et al., 2023). However, Häußler et al. (2023) only showed significant improvements in the working memory index of the WAIS-IV after 2 years of implant use, a longer follow up than used here (Häußler et al., 2023).

4. Why is the oldest group closest to the normal scores for TMTB and coding?

TMTB times for the 75+ group were shorter and closer to normal than those of the 65–74 group (Figure 1). This trend toward more normal function in this old old group is also seen in the coding scores. The reasons for this are unknown. However, the nature of the CI selection process, where older, more frail adults, with cognitive delay tend not to be referred for implantation, may well have influenced these results. Utilization data shows that uptake of CIs in the old old is particularly poor with uptake rates of less than 1% of suitable candidates (Sorkin and Buchman, 2016). Thus, only the fittest and most able older adults may come forward to seek treatment.

### Limitations

The sample was limited to those hearing-impaired adults who had been selected as suitable CI candidates. This excluded individuals with higher baseline levels of cognitive impairment or depression. The symbol coding test was the only test where a hearing test was reported to exclude those with undiagnosed hearing impairment from the sample used to calculate the normative values. Thus, normal values reported for the TMTB, TUG and MMSE may include individuals with undiagnosed hearing loss. The difference in scores between normally hearing individuals and hearing-impaired individuals may thus be greater than reported here. Instructions were given in written format, but we cannot exclude an impact of test presentation mode on scores. The follow up of 18 months was potentially not long enough to see greater changes in cognitive measures. Some of the study data was collected during a period where COVID-19 pandemic social distancing restrictions were in place, and subjects may not have been undertaking their normal activities and have had less appointments at the clinic. Although subjects all used cochlear implant devices manufactured by the same company, we expect these results to be relevant to all CI users regardless of device manufacturer.

## Conclusion

Severe to profoundly deaf individuals with hearing aids or cochlear implants were poorer than age-equivalent normally hearing individuals with respect to cognitive flexibility, attention, working memory, processing speed and visuoperceptual functions. The cochlear implant improved executive function as measured in the symbol coding test in the 60–64 age group. The coding task relies to a lesser extent on working memory than the TMTB, but requires good manual dexterity, attention and processing speed.

We suggest that the greatly improved hearing provided by the cochlear implant likely improved attention and processing speed, as a consequence of reallocation of cognitive resources away from auditory processing and back to cognitive tasks in the youngest age group.

The oldest age group of participants had mean standardized cognition scores closest to normal values, suggesting that only the most able older seniors tend to come forward for a CI.

Further research is required into the effects of severe to profound hearing loss on cognition and should focus on specific areas of cognitive function as in this paper, moving away from more generalized screening tests such as the MMSE.

## Data Availability

Deidentified individual participant data and relevant study documentation used in this study are available upon reasonable written request to author CJJ (cjames@cochlear.com).
