# Supplementary material for: Cognitive processing speed improvement after cochlear implantation
Source: Front Aging Neurosci. 2024 Sep 17;16:1444330. doi: 10.3389/fnagi.2024.1444330 (PMC11442269; doi:10.3389/fnagi.2024.1444330)
Supplement: Supplementary file 1 [file Table_1.DOCX]

**Baseline WAIS-IV age-referenced coding score**

AGE.group n m sd l u

(59,65] 19 5.21 2.92 1 10

(65,75] 43 7.40 4.32 1 19

(75,93] 29 9.66 4.95 3 19

**12months WAIS-IV age-referenced coding score**

AGE.group n m sd l u

(59,65] 18 6.94 4.49 1 17

(65,75] 37 7.68 4.80 1 19

(75,93] 26 8.46 4.64 1 18

**18months WAIS-IV age-referenced coding score**

AGE.group n m sd l u

(59,65] 18 7.39 4.82 1 19

(65,75] 36 7.94 5.00 1 18

(75,93] 27 9.59 5.15 1 19

**Baseline log10(TMTB)**

AGE.group n m sd l u

(59,65] 16 2.04 0.340 1.46 2.57

(65,75] 44 2.10 0.208 1.78 2.57

(75,93] 30 2.20 0.242 1.68 2.51

**12months log10(TMTB)**

AGE.group n m sd l u

(59,65] 16 1.99 0.205 1.52 2.32

(65,75] 36 2.04 0.202 1.58 2.44

(75,93] 24 2.10 0.299 1.34 2.56

**18months log10(TMTB)**

AGE.group n m sd l u

(59,65] 16 1.94 0.187 1.51 2.18

(65,75] 32 2.06 0.216 1.54 2.56

(75,93] 26 2.17 0.258 1.72 2.67

**Baseline log10(31-MMSE)**

AGE.group n m sd l u

(59,65] 20 0.425 0.372 0 0.954

(65,75] 45 0.506 0.304 0 1

(75,93] 31 0.588 0.320 0 1.15

**12months log10(31-MMSE)**

AGE.group n m sd l u

(59,65] 19 0.485 0.379 0 0.954

(65,75] 38 0.511 0.320 0 1.04

(75,93] 27 0.482 0.326 0 1

**18months log10(31-MMSE)**

AGE.group n m sd l u

(59,65] 19 0.458 0.316 0 0.954

(65,75] 36 0.457 0.320 0 1.04

(75,93] 28 0.555 0.272 0 1.04

**Baseline log10(TUG)**

AGE.group n m sd l u

(59,65] 20 0.958 0.141 0.778 1.23

(65,75] 45 1.03 0.183 0.477 1.81

(75,93] 32 1.06 0.186 0.602 1.54

**12months log10(TUG)**

AGE.group n m sd l u

(59,65] 19 0.963 0.153 0.699 1.30

(65,75] 38 0.984 0.122 0.699 1.28

(75,93] 28 1.07 0.170 0.845 1.49

**18months log10(TUG)**

AGE.group n m sd l u

(59,65] 19 0.948 0.130 0.699 1.20

(65,75] 35 0.988 0.118 0.699 1.23

(75,93] 28 1.05 0.195 0.602 1.52
